# Placental Malaria is Associated with Higher LILRB2 Expression in Monocyte Subsets and Lower Anti-Malarial IgG Antibodies During Infancy

**DOI:** 10.3389/fimmu.2022.909831

**Published:** 2022-07-12

**Authors:** Celia Dechavanne, Odilon Nouatin, Rafiou Adamou, Sofie Edslev, Anita Hansen, Florian Meurisse, Ibrahim Sadissou, Erasme Gbaguidi, Jacqueline Milet, Gilles Cottrell, Laure Gineau, Audrey Sabbagh, Achille Massougbodji, Kabirou Moutairou, Eduardo A. Donadi, Edgardo D. Carosella, Philippe Moreau, Ed Remarque, Michael Theisen, Nathalie Rouas-Freiss, André Garcia, Benoit Favier, David Courtin

**Affiliations:** ^1^ UMR 261 MERIT, Université Paris Cité, Institut de Recherche pour le Développement (IRD), Paris, France; ^2^ Centre d’Etude et de Recherche sur le Paludisme Associé à la Grossesse et à l’Enfance, Cotonou, Benin; ^3^ Centre for Medical Parasitology at Department of International Health, Immunology and Microbiology, University of Copenhagen, Copenhagen, Denmark; ^4^ Center for Immunology of Viral, Auto-immune, Hematological and Bacterial diseases (IMVA-HB/IDMIT), Université Paris-Saclay, Inserm, CEA, Fontenay-aux-Roses, France; ^5^ Laboratoire de Biologie et Physiologie Cellulaires, Faculté des Sciences et Techniques, Université d’Abomey-Calavi, Cotonou, Benin; ^6^ Laboratory of Clinical Immunology, Ribeirão Preto Medicine School, University of São Paulo, Ribeirão Preto, Brazil; ^7^ CEAA, DRF-Institut François Jacob, Service de Recherches en Hémato-Immunologie, Hôpital Saint-Louis, Paris, France; ^8^ U976 HIPI Unit, IRSL, Université Paris, Paris, France; ^9^ Department of Parasitology, Biomedical Primate Research Centre, Rijswijk, Netherlands; ^10^ Department for Congenital Disorders, Statens Serum Institut, Copenhagen, Denmark

**Keywords:** LILRB1, LILRB2, *Plasmodium falciparum*, immune tolerance, HLA-G, Gd T cell, monocytes, malaria candidate vaccine

## Abstract

**Background:**

Placental malaria (PM) is associated with a higher susceptibility of infants to *Plasmodium falciparum (Pf)* malaria. A hypothesis of immune tolerance has been suggested but no clear explanation has been provided so far. Our goal was to investigate the involvement of inhibitory receptors LILRB1 and LILRB2, known to drive immune evasion upon ligation with pathogen and/or host ligands, in PM-induced immune tolerance.

**Method:**

Infants of women with or without PM were enrolled in Allada, southern Benin, and followed-up for 24 months. Antibodies with specificity for five blood stage parasite antigens were quantified by ELISA, and the frequency of immune cell subsets was quantified by flow cytometry. LILRB1 or LILRB2 expression was assessed on cells collected at 18 and 24 months of age.

**Findings:**

Infants born to women with PM had a higher risk of developing symptomatic malaria than those born to women without PM (IRR=1.53, p=0.040), and such infants displayed a lower frequency of non-classical monocytes (OR=0.74, p=0.01) that overexpressed LILRB2 (OR=1.36, p=0.002). Moreover, infants born to women with PM had lower levels of cytophilic IgG and higher levels of IL-10 during active infection.

**Interpretation:**

Modulation of IgG and IL-10 levels could impair monocyte functions (opsonisation/phagocytosis) in infants born to women with PM, possibly contributing to their higher susceptibility to malaria. The long-lasting effect of PM on infants’ monocytes was notable, raising questions about the capacity of ligands such as Rifins or HLA-I molecules to bind to LILRB1 and LILRB2 and to modulate immune responses, and about the reprogramming of neonatal monocytes/macrophages.

## Introduction

Each year, up to 125 million pregnancies occur in malaria endemic countries, with a heightened risk of poor outcomes including miscarriage, maternal death and severe anaemia (1, 2). Pregnancy-associated malaria is also deleterious for the newborn (3), leading to low birth weight and increasing the risk of infant morbidity and mortality (1). Placental malaria (PM) due to *Plasmodium falciparum (Pf)* is estimated to cause up to 100,000 infant deaths every year (4). Children born to mothers with PM are more susceptible to malaria (3). PM shortens the delay to first malaria infection (5–9), and this is thought to be due to a phenomenon named immune tolerance (IT). This phenomenon may be driven by fetal sensitization to malaria antigens *in utero* leading to a modification of immune development of the foetus (10, 11). At present, no unequivocal explanation has been proposed.

Leukocyte immunoglobulin like receptor B (LILRB)1 and LILRB2 are inhibitory receptors that play an important role in the regulation of immune responses that modulate progression or control of infectious diseases (12, 13). LILRB2 is exclusively expressed by myeloid cells including monocytes, dendritic cells and neutrophils (12). In contrast, LILRB1 is found on monocytes and dendritic cells but also on B cells and subsets of CD8 T, γδ T and NK cells (12). Binding of LILRB1 and LILRB2 to HLA-I molecules affects the function of the corresponding immune cell populations, thereby modulating crucial steps in the immune response such as cell differentiation, migration, proliferation, cytotoxicity and cytokines or antibody production. Recent studies indicate a complex interplay between monocytes and malaria infection. Indeed, monocytes play an important role in the immune responses against malaria through phagocytosis and cytokine production. However, exacerbated activation of monocytes could also increase the level of inflammation, leading to detrimental host immune responses (14). The non-classical HLA class I molecule HLA-G, known to be involved in maternal maternofetal tolerance, presents the highest affinity for binding to LILRB1 and LILRB2. HLA-G and IL10 mutually up-regulate their expression, and are involved in neonatal immunoregulatory mechanisms (15). Of note, high plasma levels of HLA-G were previously associated with an increased risk of *Pf* infection in infants (16, 17).

In the present study, we aimed to define the role of PM on immune profiles as well as on the level of anti-malarial antibodies in infant with a particular focus on LILRB1, LILRB2, IL-10 and HLA-G expression. These data bring a better understanding of the dynamics of monocyte subsets and LILRB1/LILRB2 inhibitory receptor expression during *Pf* infection with potential implications for the design of new therapeutic strategies against malaria.

## Methods

### Study Design and Follow-up

The present follow-up is part of a study concerning 1,183 pregnant women participating in *Malaria in Pregnancy Preventive Alternative Drugs*, a randomized trial of intermittent preventive treatment (IPTp) in Benin (18). The first 154 infants for which flow cytometry data were available were enrolled from January 2010 to June 2011 and followed throughout the first 2 years of life in the TOLIMMUNPAL project (19). At delivery, active placental malaria was determined by impression smear from the placental blood stained with Giemsa. At birth, newborn’s gender, weight and axillary temperature were recorded. At 6, 9, 12, 18 and 24 months, a medical questionnaire was filled-out. A thick blood smears (TBS) was performed monthly to detect asymptomatic malaria carriage. A rapid diagnosis test (RDT) for malaria was performed during the follow-up if an infant was febrile. Fever was defined as an axillary temperature greater or equal to 37.5°C. Symptomatic malaria was defined as a combination of a malaria infection determined by a positive RDT and/or a positive TBS and the presence of fever (or a 24 hours’ history of fever) since infants are particularly vulnerable to symptomatic malaria. However, we cannot exclude that non-malaria infections can also participate to the observed fever in case of coinfection. Malaria attacks were treated with artemether-lumefantrine combination, as recommended by the Beninese National Malaria Control Program. An asymptomatic infection was defined as a positive monthly systematic TBS with no fever or no history of fever.

The design of the study follow-up children for 2 years based on two arguments. First, epidemiological investigations from our group demonstrated that the risk of first *Plasmodium falciparum* infection associated with placental malaria is observed during the first 2 years of life (5, 20). Second, Malhotra et al, demonstrated the association between placental malaria and the modulation of T cell responses in children up to 3 years of age (10). Important modulation of T cell responses related to exposed not sensitized (putatively tolerant) children were observed at 18 and 24 months of age in this study. These findings led us to select these two time points for assessment of LILIRB1 and LILRB2 expression in immune cell populations. Therefore, peripheral blood at 18 and 24 months was collected to perform the cellular immune cell phenotyping including LILRB1 and LILRB2 expression, to determine antibodies, cytokines and HLA-G concentrations.

We previously showed the importance to take into account the local malaria transmission to demonstrate the immune tolerance hypothesis (20). In the present study, Center for Disease Control light traps were used on two consecutive nights per month between April 2011 and February 2013. The traps were installed at dark and collected at dusk in 180 of the 400 infant’s bedrooms. The selection of 180 bedrooms was made on the basis of geographical equidistribution criteria. All mosquitoes were brought to the laboratory for identification (genus and species) and all Anopheles mosquitoes were kept individually for further analysis (21). Climatic factors as well as household characteristics were recorded throughout the study in all household. The following information was systematically collected: soil type, vegetation index, proximity of a watercourse, type of roof, number of windows and doors, number of inhabitants, ownership of a bed-net or insect repellent, presence of abandoned objects susceptible to be used as oviposition sites for female mosquitoes and presence of areas where building constructions are ongoing with tools or holes representing potential breeding habitats for anopheles. From these data, a predictive regression model was developed to predict the spatiotemporal malaria exposure risk for each infant (22).

### Antibody, Cytokine and HLA-G Measurements

The Enzyme Linked ImmunoSorbent Assay (ELISA) standard operating procedures developed by the African Malaria Network Trust was used to assess antibody concentrations to MSP1, MSP2, MSP3, AMA1 and GLURP antigens. All details about the recombinant proteins and the procedure are described by Dechavanne et al. (23).

Concentrations of IL-4, IL-10, TNF-α and IFN-γ were determined using BD OptEIA ELISA Set (BD Biosciences, San Diego, CA, USA) following the manufacturer’s protocol and as previously described by our team (24). The levels of soluble HLA-G isoforms HLA-G1 and HLA-G5 were determined by ELISA assay as we described previously (17).

### Immune Cell Phenotyping and LILRB1 and LILRB2 Expression

**Figure 1:**
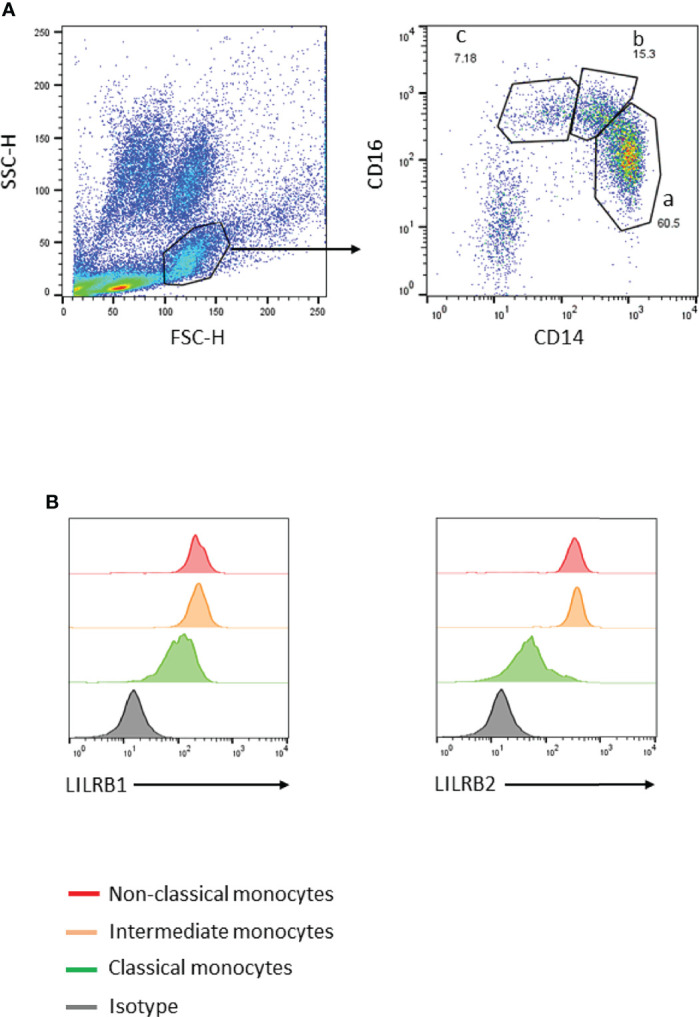
Gating strategy to identify peripheral blood monocyte subsets. **(A)** Monocytes were gated using forward- and side-scatter properties. The 3 subsets were characterized through expression of CD14 and CD16 markers (a: classical CD14^++^CD16^–^, b: intermediate CD14^++^CD16^+^, and c: non-classical CD14^+^CD16^++^). **(B)** LILRB1 or LILRB2 expression levels on the 3 monocyte subsets were defined using histogram geometric mean.

Phenotype analysis was performed on infant peripheral blood (1 mL) the day of collection. The expression of LILRB1 and/or LILRB2 inhibitory receptors on the surface of the following cell populations was analyzed: T helper cells (CD3^+^CD4^+^), T cytotoxic cells (CD3^+^CD8^+^), T regulatory cells (CD3^+^CD4^+^CD25^high^CD127^-^), T effector cells (CD3^+^CD4^+^CD25^+^CD127^+^), γδ T cells (CD3^+^Vd2^+^) and B cells (CD19^+^). NK cell subsets were characterized by expression of CD56 and CD16 among the mononuclear lymphoid cells. Blood monocyte subpopulations were characterized by expression of CD14 and CD16 markers in the population of mononuclear cells. Neutrophils and eosinophils are respectively CD16^+^ and CD16^-^ in the polymorphonuclear cell population. A classical gating strategy was used to investigate monocyte dysregulation during *Plasmodium falciparum* infection in children. A second analysis by contour plot was also applied to validate the gates of our flow cytometry analysis for all samples. Due to the inter-individual variability in the proportions of monocyte populations or the variations induced by parasite infection, the selection of monocyte subsets was adapted for each sample. To define positive and negative events, isotype-matched control antibodies were used. Flow cytometer data were analyzed using FlowJo software version 10. The gating strategy is presented in **Figure 1**.

### Ethics

The study was conducted in the context of the MiPPAD “Malaria in Pregnancy Preventive Alternative Drugs,” (http://clinicaltrials.gov/ct2/show/NCT00811421) and TOLIMMUNPAL programs. Both projects were approved by the Ethics Committee of the Faculté des Sciences de la Santé de Cotonou. The TOLIMMUNPAL study protocol and informed consent were also approved by the Comité Consultatif de Déontologie et d’Éthique (CCDE) of the Institut de Recherche pour le Développement (IRD, France).

### Statistics

Confounders were environmental exposure (22), infant sex, infant age, birth weight, health centre, ethnic group, maternal anemia and maternal IPTp. A Chi-square test was performed to assess the differences between maternal or neonatal characteristics. The risk of malaria infection between 18 and 24 months of age was compared between infants born to mother with and without PM using the total number of *Pf* infections during the 6-month period and a negative binomial regression adjusted on confounders.

Univariate analysis was performed at 18 and 24 months of age using a linear regression to assess (i) the effect of PM on the frequency of immune cell populations and on LILRB1 and LILRB2 expression and (ii) the effect of active malaria infection on LILRB1 and LILRB2 expression, on cytokines and HLA-G levels.

For multivariate analysis, a mixed linear model was used, taking into account the measurement at 18 and 24 months of age together in one analysis. The effect of PM or of an active malaria infection was assessed on the frequency of monocytes, LILRB1 and LILRB2 expression, cytokines, HLA-G and anti-malarial antibody concentrations. All models were adjusted on potential confounders and on the frequency of each subset of immune cell in the LILRB1 and LILRB2 expression analyses. For antibody and cytokines levels analysis, an interaction term was included to determine if the effect of an active malaria infection at the moment of blood draw depends on whether the infant is born to mother with or without PM. Data were analysed with Stata^®^ Software, Version 13 (StatCorp LP, College Station, TX, USA) and the graphs were done using Graph Pad Prism (Version 8.1.2).

### Role of Funding Sources

Funders had no role in study design; collection, analysis and interpretation of data; writing of manuscript; and in the decision to submit the paper for publication.

### Data Availability

Raw experimental data associated with the figures presented in the manuscript are available from the corresponding author upon reasonable request.

## Results

### Infants Born to Mothers with PM Have a Higher Risk of Symptomatic Malaria

The objective of this study was to investigate the immune response at 18 and 24 months of age in infants born to mothers with or without PM. None of the main characteristics of the studied population were found to differ between infants born to mothers with a *Pf*-infected or an uninfected placenta (**Table 1**). However, infants born to mothers with PM had a significantly higher risk of developing symptomatic malaria (adjusted negative binomial regression IRR=1.533; [1.020;2.305], p=0.04) between 18 and 24 months of age. There were no differences in the numbers of asymptomatic infections between the two groups of infants.

**Table 1:** Characteristics of the population.

	Negative placental infection*	Positive placental infection*	
	n (%)	n (%)	*p-value*
**Maternal characteristics**			
Ethnic group			0,777
Fon	30 (23.62)	8 (29.63)	
Aizo	84 (66.14)	16 (59.26)	
Others	13 (10.24)	3 (11.11)	
Gravidity status			0,415
Primigravidity	107(84.25)	21 (77.78)	
Multigravidity	20 (15.75)	6 (22.22)	
Malaria prophylaxis			0,673
SP	48 (37.8)	8 (29.63)	
MQSD	37 (29.13)	8 (29.63)	
MQFD	42 (33.07)	11 (40.74)	
**Neonatal characteristics**			
Gender			0,645
Male	55 (43.31)	13 (48.15)	
Female	72 (56.69)	14 (51.85)	
Birth weight			0,811
< 2500	12 (9.6)	3 (11.11)	
≥ 2500	113 (90.4)	24 (88.89)	

*Active placental malaria was determined by impression smears from the placental blood. The maternal and neonatal characteristics of the population were presented according to the presence or absence of active placental malaria. Out of 154 newborns, 27 (17.42%) were born from a mother with placental malaria. Chi-square test was performed to compare the potential confounders in presence or absence of active placental infection.

### Non-Classical Monocytes are Less Frequent in Infants Born to Mothers With PM and Highly Express LILRB2

**Figure 2:**
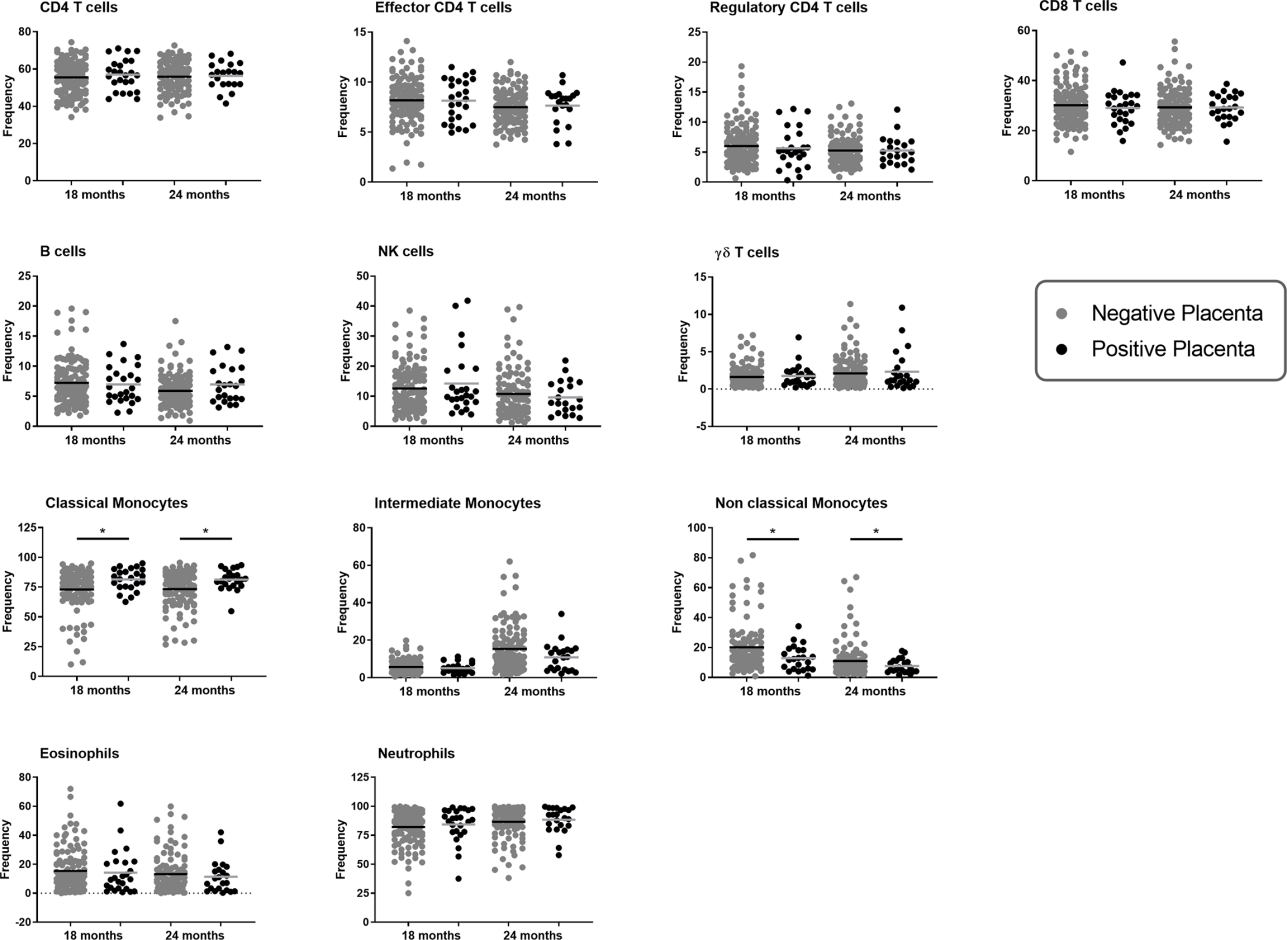
Immune cell populations at 18 and 24 months of age according to placenta malaria. The frequency of each immune cell population at either 18 or 24 months of age was represented in one graphic. Cell subset frequencies were determined as followed: CD4, CD8 and γδ T cells among total CD3^+^ T cells; regulatory and effector CD4^+^ T cells among CD4^+^ CD3^+^ T cells; Monocyte subsets among total monocytes, B or NK cells among lymphoid cells and neutrophils or eosinophils among granulocytes gated using forward- and side-scatter properties. The frequency of immune cells in infants born form mother with or without placental malaria are represented in gray and dark plain round respectively. The horizontal dark line in the beeswarm plot represents the median value. A linear regression was performed to compare the frequencies between the two groups of infants (infants born from a mother with an active placental malaria infection (n=27) vs infants born from a mother without placental malaria infection (n=127)). *: p value lower than 0.05 (and higher than 0.01).

**Figure 3:**
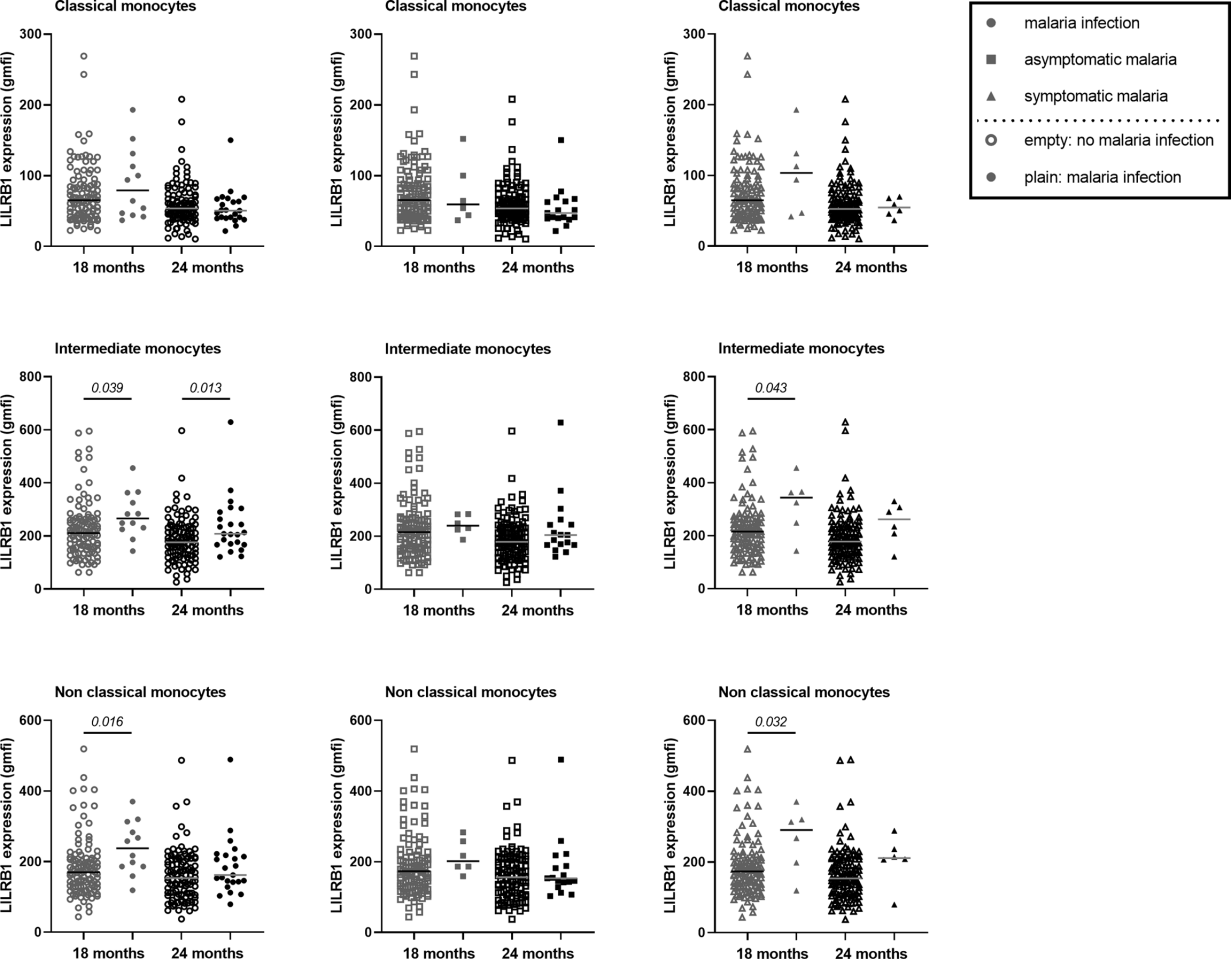
Higher expression of LILRB1 on intermediate and non-classical monocytes at 18 and 24 months of age during active malaria. The expression of LILRB1 on immune cells was measured with the geometric mean of fluorescence intensity (gmfi) between *Pf*-infected and not-infected infants at 18 or 24 months of age. Total, asymptomatic or symptomatic malaria infections were represented with plain circle, square and triangle respectively. No infection at the visit was represented with empty symbols. The horizontal dark line in the beeswarm plot represents the median value. The number of infants with total, asymptomatic or symptomatic malaria infections varies between 6 and 23 for all monocyte subtypes. A linear regression (univariate analysis) was performed to compare the expression between the two groups of infants. P values are indicated in italic.

**Figure 4:**
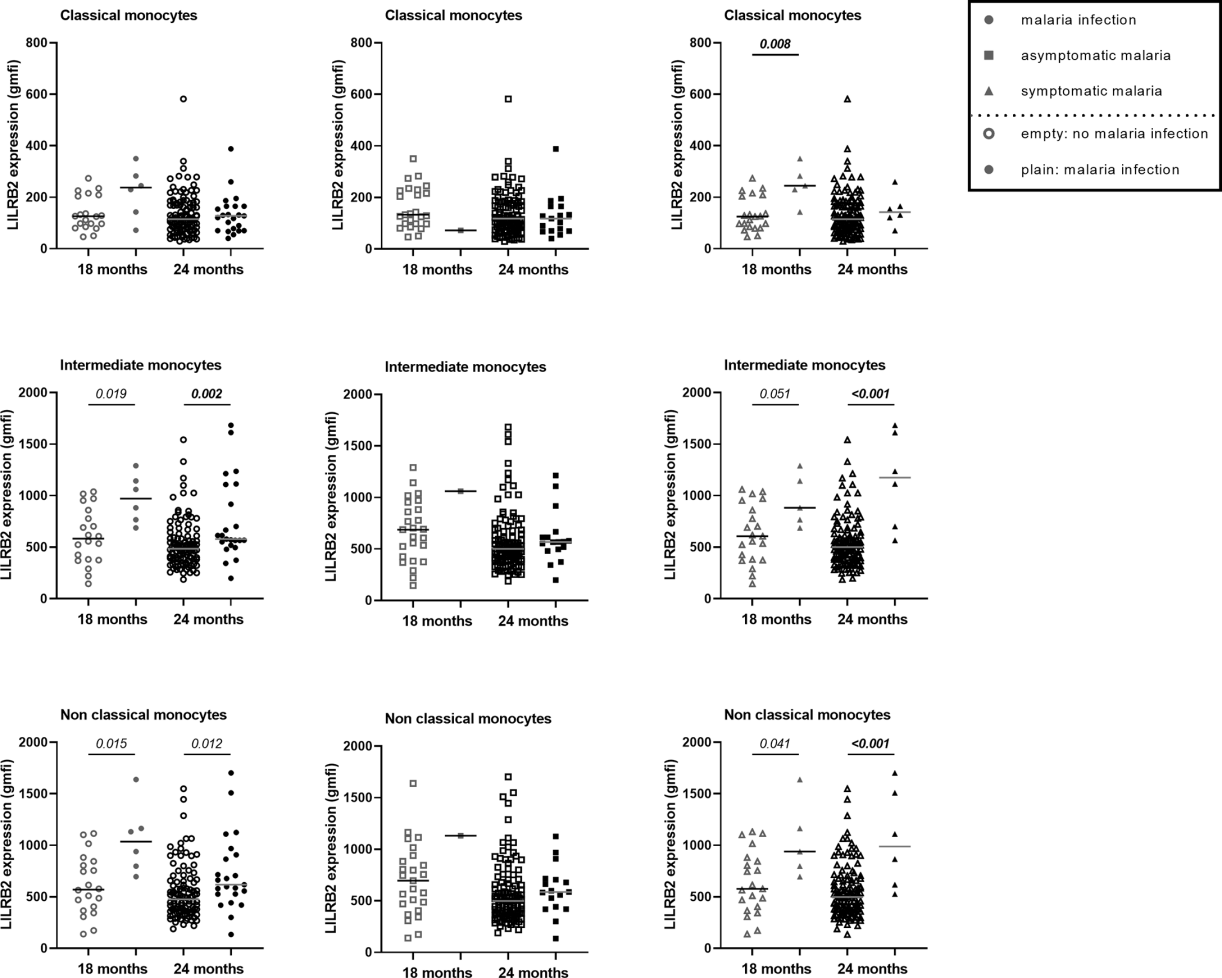
Higher expression of LILRB2 on intermediate and non-classical monocytes at 18 and 24 months during active malaria. The expression of LILRB2 on immune cells was measured with the geometric mean of fluorescence intensity (gmfi) between infected and not-infected infants at 18 or 24 months of age. Respectively 16 and 25 *Pf* infections were reported at 18 and 24 months among which 10 and 6 were symptomatic *Pf* infections. Total, asymptomatic or symptomatic malaria infections were represented with plain circle, square and triangle respectively. No infection at the visit was represented with empty symbols. The horizontal dark line in the beeswarm plot represents the median value. The number of infants with total, asymptomatic or symptomatic malaria infections varies between 1 and 23 for all monocyte subtypes. A linear regression (univariate analysis) was performed to compare the expression between the two groups of infants. P values are indicated in italic. In bold are the associations still significant after Bonferroni correction.

Lymphoid and myeloid immune cells were analyzed in order to assess the effect of PM on infant immunity at 18 and 24 months of age. To this end, a flow cytometry gating strategy was set-up to analyze the three monocyte subsets defined as classical (CD14^++^CD16^-^), intermediate (CD14^++^CD16^+^) and non-classical (CD14^+^CD16^++^) (**Figure 1**) as well as lymphoid populations, including CD4 T cells, CD8 T cells, NK cells, γδ T cells or B cells, neutrophils and eosinophils (**Figures S1**, **S2**). As shown in **Figure 2**, PM did not alter the proportions of CD4 T cells, CD8 T cells, NK cells, γδ T cells, neutrophils, eosinophils or B cells in infants. Interestingly, PM was associated with a higher frequency of classical monocytes and a lower frequency of intermediate and non-classical monocytes. In the multivariate analyses, the proportion of non-classical monocytes remained significantly lower in infants born to mothers with PM, independently of active malaria infection at the time of blood sample collection (**Table 2**). Of note, malaria infection at the time of sample collection was associated with a reduced frequency of classical monocytes and an increased frequency of intermediate and non-classical monocytes (**Table 2**). LILRB2 expression was higher on non-classical monocytes, and enhanced to a lesser extent on intermediate monocytes in infants born to mothers with PM as compared to infants from mothers without PM (**Table 3**). PM did not seem to influence LILRB1 expression by either classical, intermediate or non-classical monocytes (**Table 3**). Active malaria infection at the time of blood collection can influence LILRB1 and/or LILRB2 expression (**Figures 3**, **4**). Therefore, infants with active malaria infection at the time of blood collection were excluded from the multivariate analyses (**Table 3**).

**Table 2:** Placental malaria decreases non-classical monocyte sub-populations.

	Odds Ratio	p value	95% Confidence Interval
Classical monocytes			
*Pf* infection at sample collect	0,781	**<0.001**	[0.711; 0.858]
*Pf* infection in placenta	1,146	**0,013**	[1.029; 1.275]
Intermediate monocytes			
*Pf* infection at sample collect	1,383	**0,007**	[1.090; 1.755]
*Pf* infection in placenta	0,838	0,113	[0.675; 1.042]
Non classical monocytes			
*Pf* infection at sample collect	1,559	**<0.001**	[0.581; 1.999]
*Pf* infection in placenta	0,735	**0,010**	[0.581; 0.930]

Adjusted mixed models were used to assess the role of placental malaria on the frequency of monocyte sub-populations at 18 and 24 months of age for 146 infants. Mixed models are used to take into account repetitive measurements for a same individual (dependent variable). For each monocyte sub-population, one model was performed taking into account active Pf infection at the sample collection. Respectively 16 and 25 Pf infections were reported at 18 and 24 months among which 10 and 6 were symptomatic Pf infections. The model was adjusted on age, gender, birth weight, maternity, ethnicity, maternal anemia, maternal IPTp and environmental exposure. The lincom command (Stata^®^ Software, Version 13 (StatCorp LP, College Station, TX, USA)) was used to compute coefficient values in odds ratios (OR). OR > 1 means that the frequency of monocyte sub-population increases while the frequency of monocyte sub-population decreases if OR<1. Significant differences are marked in bold (p<0.05).

**Table 3:** LILRB1 and LILRB2 expression on monocyte sub-populations in infants born to PM-mothers.

	Number	Odds Ratio	p value	95% Confidence Interval
Classical monocytes				
LILRB1 expression in absence (reference) or presence of PM	142	1,073	0,448	[0.895; 1.287]
LILRB2 expression in absence (reference) or presence of PM	114	1,112	0,405	[0.866; 1.429]
Intermediate monocytes				
LILRB1 expression in absence (reference) or presence of PM	142	1,089	0,328	[0.919; 1.292]
**LILRB2 expression in absence (reference) or presence of PM**	**114**	**1,245**	**0,018**	**[1.038; 1.492]**
Non classical monocytes				
LILRB1 expression in absence (reference) or presence of PM	142	1,042	0,628	[0.880; 1.231]
**LILRB2 expression in absence (reference) or presence of PM**	**114**	**1,364**	**0,002**	**[1.121; 1.660]**

Adjusted mixed models were used to assess the influence of placental malaria on the LILRB1 and LILRB2 expression on monocyte sub-populations at 18 and 24 months of age. The geometric mean fluorescence intensity was the parameter used to study LILRB1 and LILRB2 expression on monocyte sub-populations. LILRB1 and LILRB2 expression A model was performed for the expression of each inhibitory receptor in the different monocyte sub-populations. The model was adjusted on monocyte subset frequency, age, gender, birth weight, maternity, ethnicity, maternal anemia, maternal IPTp and environmental exposure. Infants with active malaria infection at blood draw were excluded from the analysis. The lincom command (Stata^®^ Software, Version 13 (StatCorp LP, College Station, TX, USA)) was used to compute coefficient values in odds ratios (OR). OR > 1 means that the frequency of monocyte sub-population increases while the frequency of monocyte sub-population decreases if OR<1. A specific analysis was conducted (**Figures 3**, **4**; **Table 4**) for infants with active Pf-infection. Significant differences are marked in bold (p<0.05).

### Higher LILRB1, LILRB2 and IL-10 Expression in Infants With Active Malaria Infection

**Figure 5:**
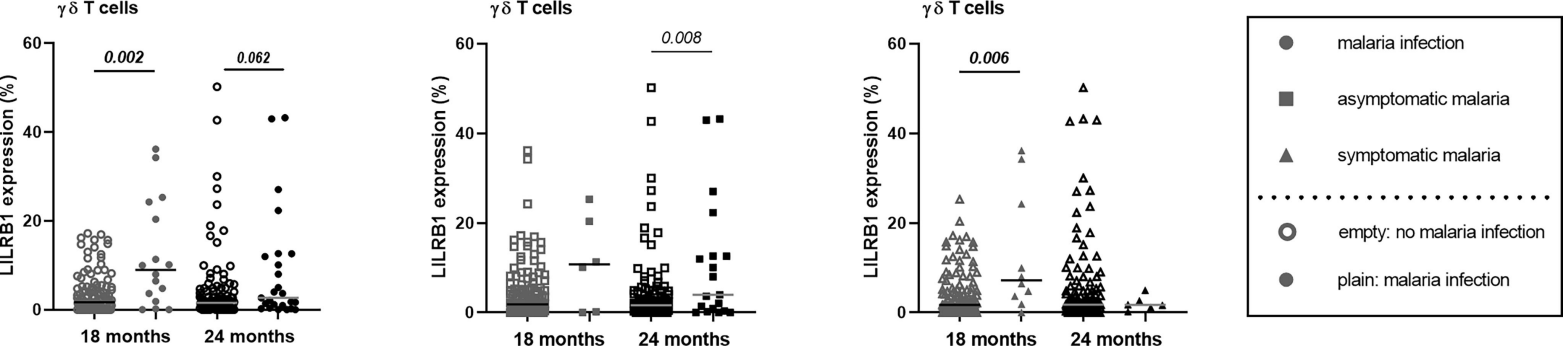
Modulation of LILRB1 expression on γδT cell surface in infant with malaria infections. The expression of LILRB1 on immune cells was measured with the geometric mean of fluorescence (gmfi) in infants at 18 and 24 months of age. Empty circle, square and triangle (gray or black) represented infant without malaria infection at the sample collect. Plain circle, square and triangle (gray or black) represented infant with malaria infection, symptomatic malaria and asymptomatic infection, respectively. The horizontal dark line in the beeswarm plot represents the median value. Respectively 16 and 25 *Pf* infections were reported at 18 and 24 months among which 10 and 6 were symptomatic *Pf* infections. P-values in bold were the one that were still considered significant after Bonferroni correction.

LILRB1 and LILRB2 receptors were expressed at higher levels on intermediate and non-classical monocytes in infants with active *Pf* infection (**Figures 3**, **4**). In a multivariate analysis, we confirmed that during active *Pf* infection, both receptors were highly expressed on intermediate and non-classical monocytes (**Table 4**) and LILRB1 expression decreased with age. Moreover, levels of LILRB1 and LILRB2 expression were higher during symptomatic attacks than during asymptomatic infections (**Figures 3**, **4**). Finally, out of all the other cell populations evaluated in this project, the levels of LILRB1 were only higher on γδ T cells in infants infected with *Pf* than in infants without malaria infection at blood draw (**Figure 5**). The multivariate analysis including the level of LILRB1 expression at 18 and 24 months confirmed that during active *Pf* infection, LILRB1 receptor was highly expressed on γδ T cell surface in infant with malaria infections (p=0.003; coef. 0.72; adjusted mixed model). A decrease of LILRB1 expression was also observed with age on γδ T cell surface in the mixed model (p=0.05; coef.=-0.051).

**Table 4:** Higher expression of LILRB1 and LILRB2 on intermediate and non-classical monocytes at 18 and 24 months during active malaria.

Independent variable	Number	Odds Ratio	p value	95% Confidence Interval
Classical monocytes				
LILRB1 expression in absence (reference) or presence of active *Pf* infection	146	1,010	0,944	[0.837; 1.212]
LILRB2 expression in absence (reference) or presence of active *Pf* infection	150	1,051	0,671	[0.820; 1.359]
Intermediate monocytes				
**LILRB1 expression in absence (reference) or presence of active *Pf* infection**	**142**	**1,299**	**0,002**	**[1.102; 1.533]**
**LILRB2 expression in absence (reference) or presence of active *Pf* infection**	**132**	**1,402**	**<0,001**	**[1.184; 1.660]**
Non classical monocytes				
**LILRB1 expression in absence (reference) or presence of active *Pf* infection**	**146**	**1,279**	**0,002**	**[1.097; 1.493]**
**LILRB2 expression in absence (reference) or presence of active *Pf* infection**	**132**	**1,362**	**0,001**	**[1.131; 1.640]**

Adjusted mixed models were used to assess the influence of Pf active infections on the LILRB1 and LILRB2 expression on monocyte sub-populations at 18 and 24 months of age. A model was performed for the expression of each inhibitory receptor in the different monocyte sub-populations. The model was adjusted on monocyte subset frequency, age, gender, birth weight, maternity, ethnicity, maternal anemia, maternal IPTp and spatiotemporal malaria exposure risk. LILRB1 expression on non-classical monocytes decreased with age in the multivariate mixed model (p>0.001; coef.=-0.052; [-0.074; -0.030]) The lincom command (Stata^®^ Software, Version 13 (StatCorp LP, College Station, TX, USA)) was used to compute coefficient values in odds ratios (OR). OR > 1 means that the frequency of monocyte sub-population increases while the frequency of monocyte sub-population decreases if OR<1. Significant differences are marked in bold (p<0.05).

**Figure 6:**
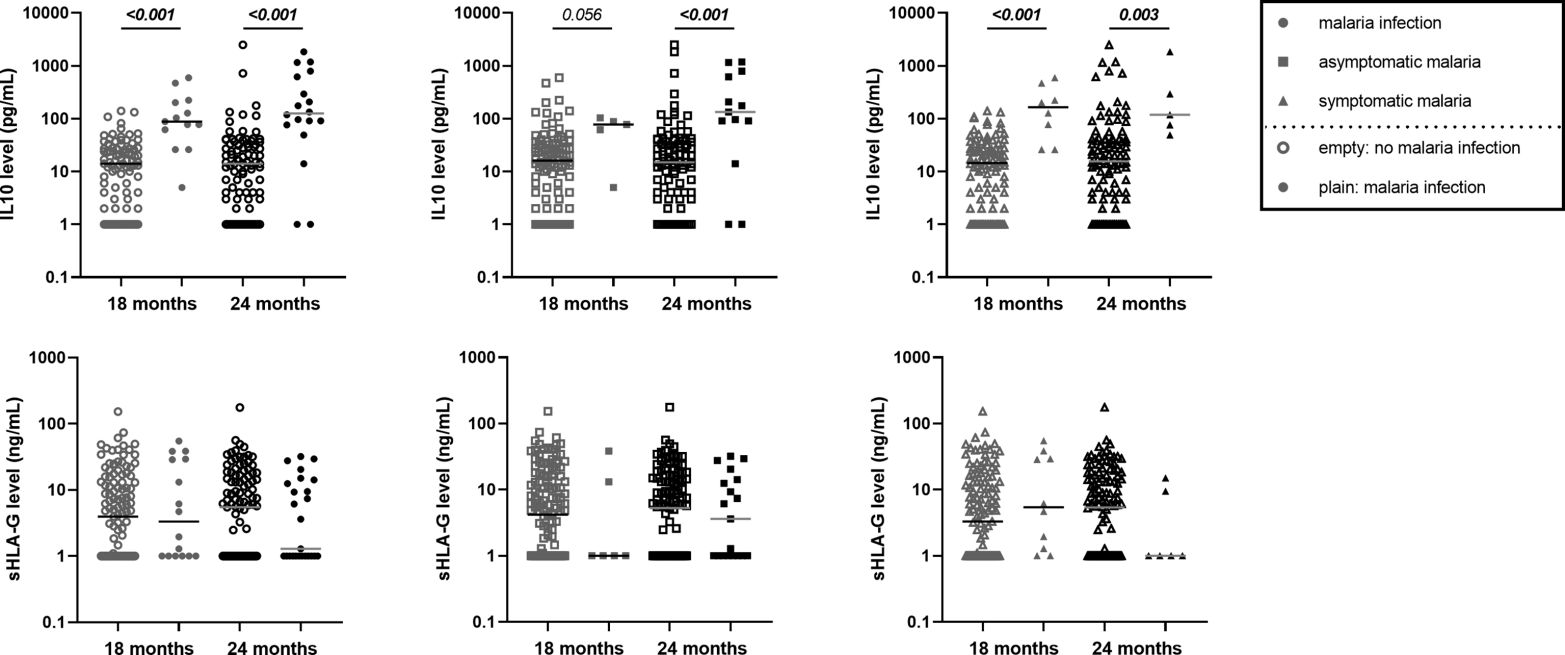
Higher IL10 level in infants with either symptomatic or asymptomatic malaria infection. The level of IL10 in pg/mL from the plasma of infant at 18 and 24 months of age was represented on the first row of graphics. HLA-G level in ng/mL from the plasma of infant at 18 and 24 months of age was represented on the second row of the graphics. Empty circle, square and triangle (gray or black) represented infants without malaria infection at the time of sample collection. Plain circle, square and triangle (gray or black) represented infant with malaria infection, symptomatic malaria and asymptomatic infection, respectively. The horizontal dark line in the beeswarm plot represents the median value. P-values in bold were the one that were still considered significant after Bonferroni correction.

At 18 and 24 months of age, cytokines (IL-4, IFN-γ, TNF-α and IL-10) and soluble HLA-G concentrations were quantified. No differences were observed as a function of PM or of active *Pf* infection except for IL-10 levels. IL-10 levels were higher in those with active malaria infections [febrile and/or asymptomatic (**Figure 6**)]. These observations were confirmed by multivariate mixed models for malaria infections (Coef=2.08; [1.587;2.579]; p<0.001), symptomatic infections (Coef=2.02; [1.455;2.950]; p<0.001) and asymptomatic infections (Coef=1.68; [0.967;2.387]; p<0.001), and mostly in infants with PM (interaction active infection*PM; Coef=2.58; [1.155;4.012]; p<0.001).

### Lower Antibody Responses in Infants Born to Mothers With PM

**Figure 7:**
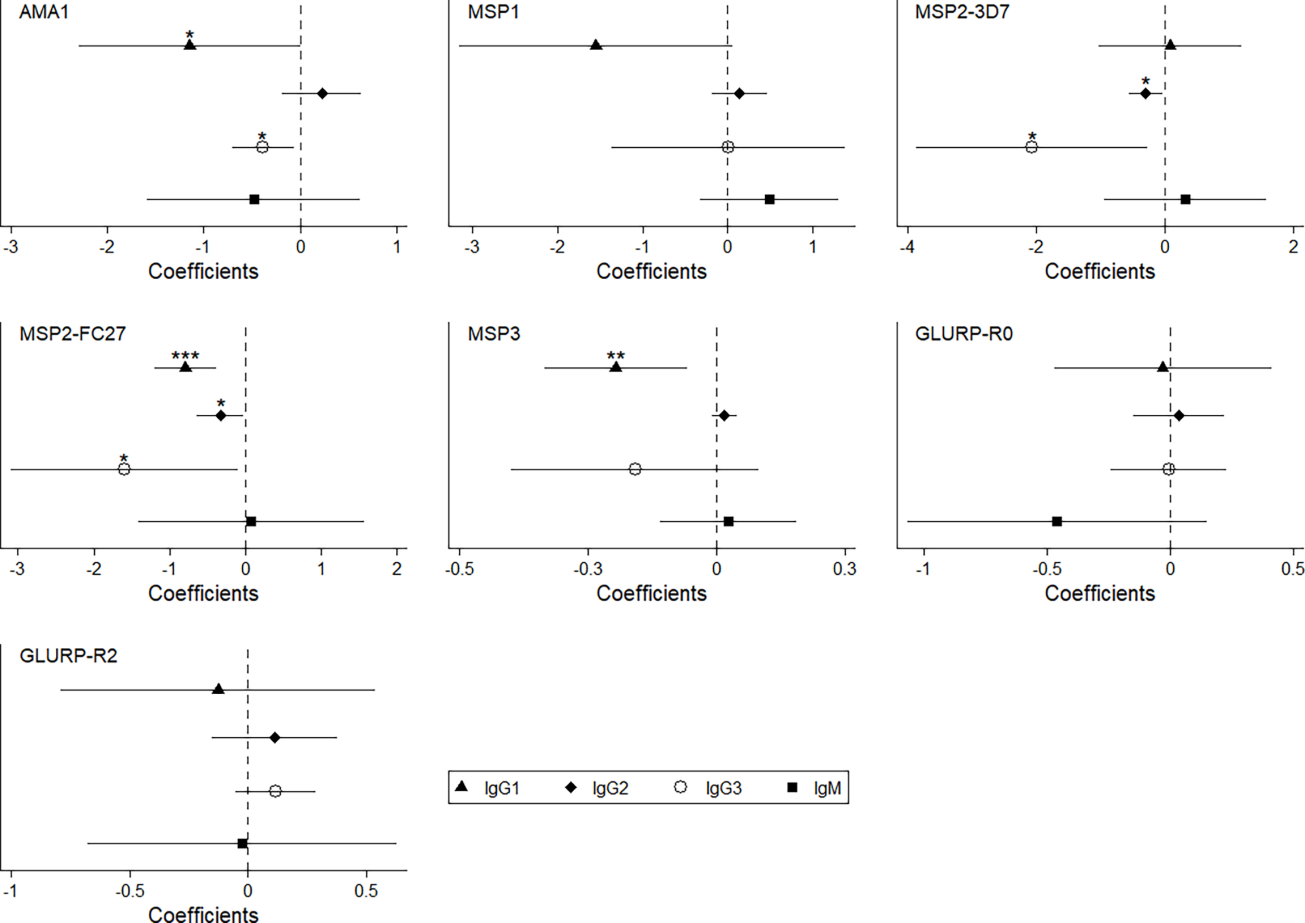
Lower level of specific anti-malarial IgG1 and IgG3 in infant born to women with PM and with malaria infection. This coefplot showed the synergic effect of PM and *Pf* active infection (interaction term) on the levels of anti-malarial antibodies in infant between 18 and 24 months of age. The models were adjusted on age, gender, birth weight, maternity, ethnicity, maternal anemia, maternal IPTp and spatiotemporal malaria exposure risk.

Higher anti-malarial antibody concentrations between 18 and 24 months of age were observed during active malaria infections (**Table 5**) mostly IgG1 and IgG3 responses against all vaccine candidate antigens (except for IgG3 to MSP1) whereas almost no IgG2 or IgM responses were affected. To understand how anti-malarial antibody levels vary in infants born from women with PM during an active *Pf* infection, we introduced an interaction term (interaction active infection*PM, **Table 5**) in the model. Infants born to mothers with PM seemed less able to mount an antibody responses during active infections (**Table 5**). Indeed, IgG1 to AMA1, MSP2 and MSP3, IgG2 to MSP2 and IgG3 to AMA1 and MSP2 were significantly lower in *Pf*-infected infants born to women with PM (**Table 5** and **Figure 7**).

**Table 5:** | Lower levels of cytophilic IgG to malaria antigens in infant born to women with PM and during active malaria.

Antibody level	*Pf* infection			PM			Interaction *Pf* infection*PM		
	Coefficient	p value	95% CI	Coefficient	p value	95% CI	Coefficient	p value	95% CI
**IgG1 to AMA1**	0.85**	**0,001**	**[0.338,1.360]**	0.84**	**0,003**	**[0.286,1.399]**	-1,15	0,050	[-2.295,0.002]
IgG2 to AMA1	-0,11	0,238	[-0.293,0.073]	-0.22*	**0,043**	**[-0.423,-0.007]**	0,22	0,281	[-0.184,0.633]
**IgG3 to AMA1**	0.30***	**<0.001**	**[0.162,0.446]**	0.22**	**0,005**	**[0.068,0.380]**	-0.38*	**0,019**	**[-0.703,-0.064]**
IgM to AMA1	0,02	0,918	[-0.359,0.399]	0,17	0,479	[-0.300,0.640]	-0,48	0,393	[-1.590,0.624]
**IgG1 to MSP1**	1.37***	**<0.001**	**[0.663,2.083]**	-0,22	0,571	[-0.974,0.537]	-1,55	0,059	[-3.157,0.058]
IgG2 to MSP1	-0,01	0,938	[-0.153,0.141]	-0,14	0,124	[-0.311,0.038]	0,14	0,390	[-0.183,0.469]
IgG3 to MSP1	0,33	0,161	[-0.129,0.779]	-0,01	0,971	[-0.600,0.578]	0,01	0,990	[-1.365,1.382]
IgM to MSP1	-0,32	0,079	[-0.676,0.038]	0,04	0,847	[-0.327,0.399]	0,50	0,231	[-0.316,1.312]
**IgG1 to MSP2-3D7**	0.58*	**0,022**	**[0.085,1.070]**	-0,27	0,336	[-0.819,0.279]	0,09	0,878	[-1.018,1.191]
**IgG2 to MSP2-3D7**	0.17***	**<0.001**	**[0.083,0.254]**	0,06	0,257	[-0.047,0.177]	-0.29*	**0,027**	**[-0.556,-0.033]**
**IgG3 to MSP2-3D7**	1.91***	**<0.001**	**[1.111,2.700]**	0,45	0,290	[-0.384,1.284]	-2.06*	**0,025**	**[-3.864,-0.258]**
IgM to MSP2-3D7	0,26	0,35	[-0.289,0.816]	0,32	0,270	[-0.245,0.879]	0,32	0,617	[-0.939,1.582]
**IgG1 to MSP2-FC27**	0.54***	**<0.001**	**[0.352,0.721]**	0.21*	**0,047**	**[0.003,0.418]**	-0.79***	**<0.001**	**[-1.201,-0.375]**
IgG2 to MSP2-FC27	0,03	0,379	[-0.041,0.107]	0,10	0,131	[-0.030,0.235]	-0.33*	**0,032**	**[-0.633,-0.028]**
**IgG3 to MSP2-FC27**	2.19***	**<0.001**	**[1.532,2.855]**	0,15	0,671	[-0.554,0.861]	-1.60*	**0,037**	**[-3.091,-0.100]**
IgM to MSP2-FC27	0,19	0,571	[-0.465,0.843]	0,33	0,328	[-0.335,1.003]	0,08	0,916	[-1.412,1.572]
**IgG1 to MSP3**	0.12**	**0,004**	**[0.038,0.197]**	0,07	0,062	[-0.004,0.147]	-0.24**	**0,006**	**[-0.402,-0.069]**
IgG2 to MSP3	0,01	0,556	[-0.010,0.018]	0,01	0,472	[-0.013,0.028]	0,02	0,194	[-0.010,0.048]
**IgG3 to MSP3**	0.30***	**<0.001**	**[0.162,0.439]**	0,08	0,195	[-0.043,0.213]	-0,19	0,199	[-0.480,0.100]
IgM to MSP3	-0,03	0,482	[-0.105,0.049]	-0,03	0,392	[-0.112,0.044]	0,03	0,724	[-0.131,0.188]
**IgG1 to GLURP-R0**	0.25*	**0,025**	**[0.031,0.460]**	0,1	0,387	[-0.128,0.331]	-0,03	0,897	[-0.470,0.411]
IgG2 to GLURP-R0	0,03	0,492	[-0.058,0.121]	0,12	0,119	[-0.031,0.273]	0,04	0,704	[-0.149,0.220]
**IgG3 to GLURP-R0**	0.17**	**0,003**	**[0.058,0.282]**	0,01	0,992	[-0.106,0.105]	-0,01	0,963	[-0.240,0.229]
IgM to GLURP-R0	0.37*	0,012	[0.083,0.666]	0.38**	**0,007**	**[0.105,0.661]**	-0,46	0,138	[-1.067,0.148]
**IgG1 to GLURP-R2**	0.56***	**0,001**	**[0.245,0.878]**	-0,04	0,815	[-0.331,0.260]	-0,13	0,709	[-0.790,0.537]
IgG2 to GLURP-R2	0,09	0,174	[-0.039,0.216]	0,09	0,149	[-0.032,0.213]	0,11	0,408	[-0.153,0.377]
**IgG3 to GLURP-R2**	0.13***	**0,001**	**[0.056,0.214]**	-0,01	0,694	[-0.084,0.056]	0,12	0,177	[-0.053,0.285]
**IgM to GLURP-R2**	0.33*	**0,039**	**[0.016,0.648]**	0,02	0,903	[-0.315,0.357]	-0,02	0,944	[-0.674,0.627]

Multivariate mixed analysis was performed to assess the association between the levels of IgG1, IgG2, IgG3 and IgM specific for seven malaria antigens and active malaria or placental malaria. An interaction term was added to the analysis to test this association in infant born from women with PM that have an active malaria infection at blood draw. The models were adjusted on age, gender, birth weight, maternity, ethnicity, maternal anemia, maternal IPTp and spatiotemporal malaria exposure risk. PM, Placental malaria; Pf, Plasmodium falciparum; 95% CI, 95% confidence interval; *p value <0.05, **p value <0.01, ***p value <0.001. Significant differences are marked in bold (p<0.05).

## Discussion

To our knowledge, this is the first study investigating LILRB1 and LILRB2 inhibitory receptor expression by circulating immune cells in the context of immune-tolerance associated with PM. Our results showed that PM affects monocyte subset frequencies, LILRB2 expression on intermediate and non-classical monocytes, and the levels of cytophilic IgG to *Pf*-merozoite antigens at 18 and 24 months of age.

Monocytes play a crucial role in controlling parasite loads and protecting the host against malaria. Their protective roles include phagocytosis, production of cytokines and antigen presentation. Monocyte-antibody cooperation also generates a major defence mechanism against malaria, known as ADCI (Antibody Dependent Cellular Inhibition) (25), in which monocytes exposed to opsonized merozoites release soluble mediators that inhibit the growth of erythrocytic parasites (26). Higher ADCI levels were associated with clinical protection against malaria (27). Collectively, these studies have emphasized the role of cytophilic IgG1 and IgG3 isotypes in immune mechanisms leading to protection from malaria infection. Their dual ability of opsonizing pathogens and binding tightly to Fc gamma receptors (28) on monocytes or neutrophils designated them as primary actors in the acquisition of natural immunity to malaria (29, 30). On one hand, our study demonstrated that infants born from women with PM had lower levels of cytophilic IgG to *Pf*-merozoite antigens between 18 and 24 months of age. On the other hand, we found a lower frequency of non-classical monocytes and a higher level of LILRB2 on those monocytes implying a capacity to modulate (attenuate) cell functions. Taken together, those data suggest impaired opsonizing phagocytic capacity in infants born from women with PM. Further studies are needed to confirm this hypothesis of an effect of PM on monocyte functions in infants.

Surprisingly, monocytes that have a relatively short half-life in the peripheral circulation (few days) were affected in 2 years old-infants by maternal PM. Our observations raise questions about the reprogramming of neonatal monocytes/macrophages in PM. Recent studies demonstrate that innate immune cells can build a “memory”. Depending on the nature of the components responsible for the first challenge, monocytes can be trained inducing non-specific attenuated or enhanced innate cell responses and functions after a second challenge (31, 32). This trained memory of human monocytes can persist for at least 3 months (33) and some protective effects have been observed up to 12 months after vaccination with BCG (34). Recent studies have shown metabolic and epigenetic changes in trained innate immune cells (35). As a result of PM, the fetal immune system can be primed *in utero* (10). Based on our observations and those recent findings (36), we could speculate that a first priming in the fetus could induce a reprogramming of the monocyte precursors reservoir which could then lead to a long-lasting tolerance to infections in infancy. In line with this, Natama et al. found that TLR7/8 stimulation of cord blood cells of neonates born to mothers with PM induced higher cytokine levels than that of neonates born to mothers without PM (37). Therefore PM could profoundly alter neonatal immune system, contributing to the higher susceptibility to infections of those infants (37, 38). Further investigations are needed to better characterize the modulation of the neonatal innate immune response (i.e. trained innate immunity) in infants born to women with PM.

Another hypothesis to explain the long-lasting effect of PM on infant monocytes could be the capability of various ligands to bind to LILRB1 and LILRB2 and consequently attenuate immune responses. We hypothesized that host molecules known for their anti-inflammatory properties could be involved in such modulation. We measured the plasma levels of IL-10 and soluble HLA-G at 18 and 24 months of age. By contrast to previous data in which higher levels of infant HLA-G were found associated with infant malaria infection during follow-up (16), here we observed no association between the levels of HLA-G in infants and active *Pf* infection at time of blood collection. However, we found a higher level of IL-10 during active malaria at both visits and for symptomatic and asymptomatic infections. The concomitant presence of IL-10 and a higher expression of LILRB1 and LILRB2 on non-classical monocytes suggests a down regulation of the cells involved in inflammatory mechanisms and therefore a potentially decreased ability to protect the infants from malaria. Moreover, *in vitro* studies indicate that IL-10 enhances the expression of LILRB2 on monocytes (39). Therefore, the increased expression of LILRB2 on monocytes could result from the production of IL-10 observed in *P. falciparum* infected individuals. These results need to be confirmed by other studies.

The recently described complex between LILRB1 and the parasite’s family of RIFIN proteins (40) may also be involved in the modulation of monocytes during malaria infections. Indeed, given their strong capacity to attenuate immune response, LILRB1 and LILRB2 represent potential targets for pathogens to evade immune recognition, thereby extending the duration of infection. Indeed, a subset of the RIFINs expressed by *Pf* were shown to bind to LILRB1 and thus lead to the inhibition of B-cell and NK cell functions (41) and to LILRB2 (42). Our data show an increase of LILRB1 and LILRB2 expression on the non-classical monocyte subsets during malaria infection and in infants born to women with PM suggesting that monocytes expressing high levels of LILRB1 and LILRB2 could be more prone to the attenuation of their immune functions possibly through the binding of the RIFINs. The long-lasting tolerance during infancy might be associated with repeated infections involving this particular subset of RIFINs. Further studies are now required to confirm those hypotheses.

Finally, δγ T cells play an important role in the immune responses against *Pf* through the production of IFN-γ that is strongly attenuated by LILRB1 engagement (43). Our data show that *Pf*-infection induced an increased frequency of δγ T cells expressing LILRB1. Similarly, to monocyte subsets, LILRB1^+^δγ T cells could also be inhibited by RIFINs or MHC-I ligands during infection leading to decreased IFN-γ production and cytotoxic activity.

In conclusion, our study provides non-exclusive scenarios for a role of PM in the development of infant immunity to malaria. Interestingly, the role of LILRB inhibitory receptors highlighted in this study raises questions on immune cell regulation pathways and the role of host or pathogen ligands. The impact of such mechanisms would help understand the mechanisms of the natural acquisition of immunity and could therefore contribute to the design of successful vaccine strategies.

## Data Availability Statement

The raw data supporting the conclusions of this article will be made available by the authors, without undue reservation.

## Ethics Statement

The studies involving human participants were reviewed and approved by Ethics Committee of the Faculté des Sciences de la Santé de Cotonou. Written informed consent to participate in this study was provided by the participants’ legal guardian/next of kin.

## Author Contributions

AM, KM, ED, EC, PM, ER, MT, NR-F, AG, BF, and DC designed research study; ON, RA, SE, AH, IS, and EG conducted experiments; ON, RA, IS, JM, GC, and DC participated to the sample collection and supervised the field study; CD, ON, FM, JM, GC, LG, AS, and DC analysed the data. CD, ON, BF, and DC drafted the first version of the manuscript. All authors read and approved the final manuscript.

## Funding

This study was supported by the Agence Nationale de la Recherche (ANR). Two PhD scholarships were awarded by IRD and the French Embassy, Cotonou to IS and RA. The clinical trial in which the study was nested was funded by the European Developing Countries Clinical Trials Partnership (EDCTP; IP.2007.31080.002). This work was also supported by the Brazil-France research cooperation program USP/COFECUB (grant no. Uc Me 169-17).

## Conflict of Interest

The authors declare that the research was conducted in the absence of any commercial or financial relationships that could be construed as a potential conflict of interest.

## Publisher’s Note

All claims expressed in this article are solely those of the authors and do not necessarily represent those of their affiliated organizations, or those of the publisher, the editors and the reviewers. Any product that may be evaluated in this article, or claim that may be made by its manufacturer, is not guaranteed or endorsed by the publisher.

## Supplementary Material

Supplementary Figure 1 Gating strategy to define proportions of peripheral blood lymphoid subsets. Lymphocytes were gated using forward- and side-scatter properties. Characterization of T helper cells (CD3^+^CD4^+^), CD8 T cells (CD3^+^CD8^+^), T regulatory cells (CD3^+^CD4^+^CD25^high^CD127^-^), T effector cells (CD3^+^CD4^+^CD25^+^CD127^+^), γδ T cells (CD3^+^Vd2^+^) and B cells (CD19^+^). NK cell subsets were determined based on the expression of CD56 and CD16 markers (CD56^mid^CD16^-^, CD56^dim^CD16^+^, CD56^high^CD16^-^).

Supplementary Figure 2 Gating strategy to characterize proportions of peripheral blood neutrophils and eosinophils. Granulocytes were gated using forward- and side-scatter properties. Neutrophils were defined as CD16^+^ cells and eosinophils as CD16- cells.
