# Supplementary figures and images for: Placental Malaria is Associated with Higher LILRB2 Expression in Monocyte Subsets and Lower Anti-Malarial IgG Antibodies During Infancy
Source: Front Immunol. 2022 Jul 12;13:909831. doi: 10.3389/fimmu.2022.909831 (PMC9326509; doi:10.3389/fimmu.2022.909831)

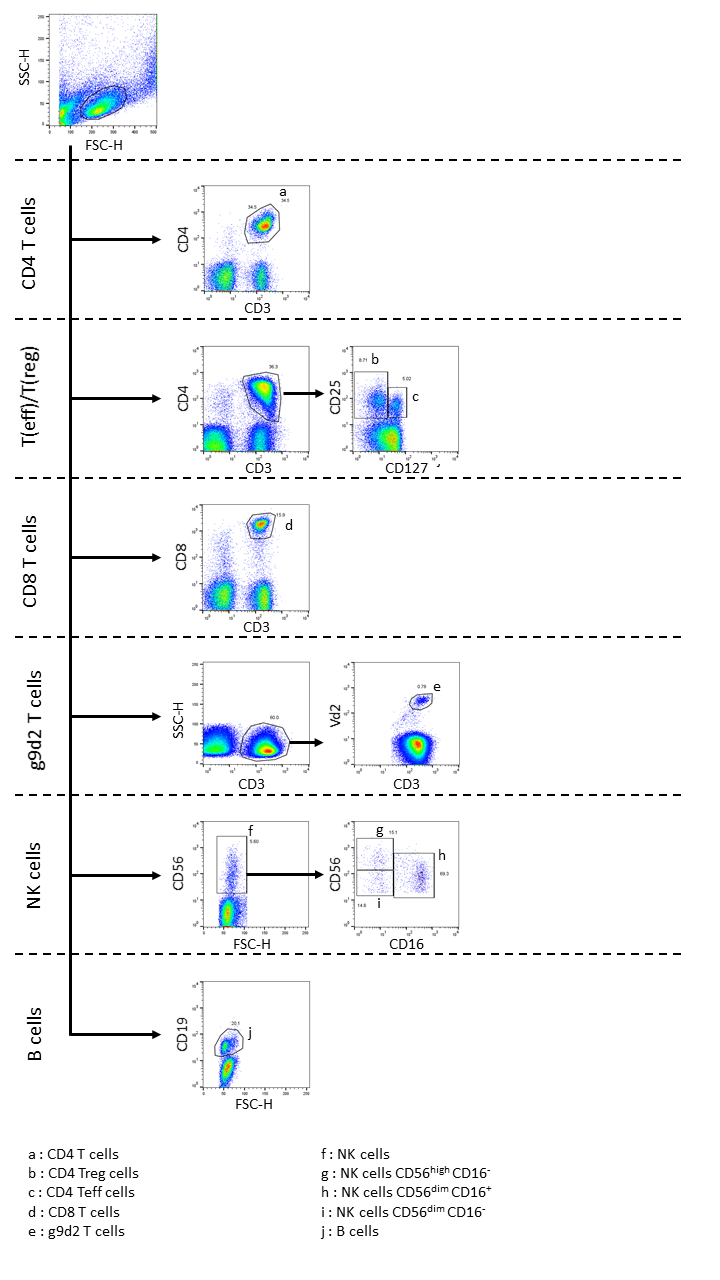

Supplement: Supplementary Figure 1 — Gating strategy to define proportions of peripheral blood lymphoid subsets. Lymphocytes were gated using forward- and side-scatter properties. Characterization of T helper cells (CD3+CD4+), CD8 T cells (CD3+CD8+), T regulatory cells (CD3+CD4+CD25highCD127-), T effector cells (CD3+CD4+CD25+CD127+), γδ T cells (CD3+Vd2+) and B cells (CD19+). NK cell subsets were determined based on the expression of CD56 and CD16 markers (CD56midCD16-, CD56dimCD16+, CD56highCD16-). [file Image_1.tif]

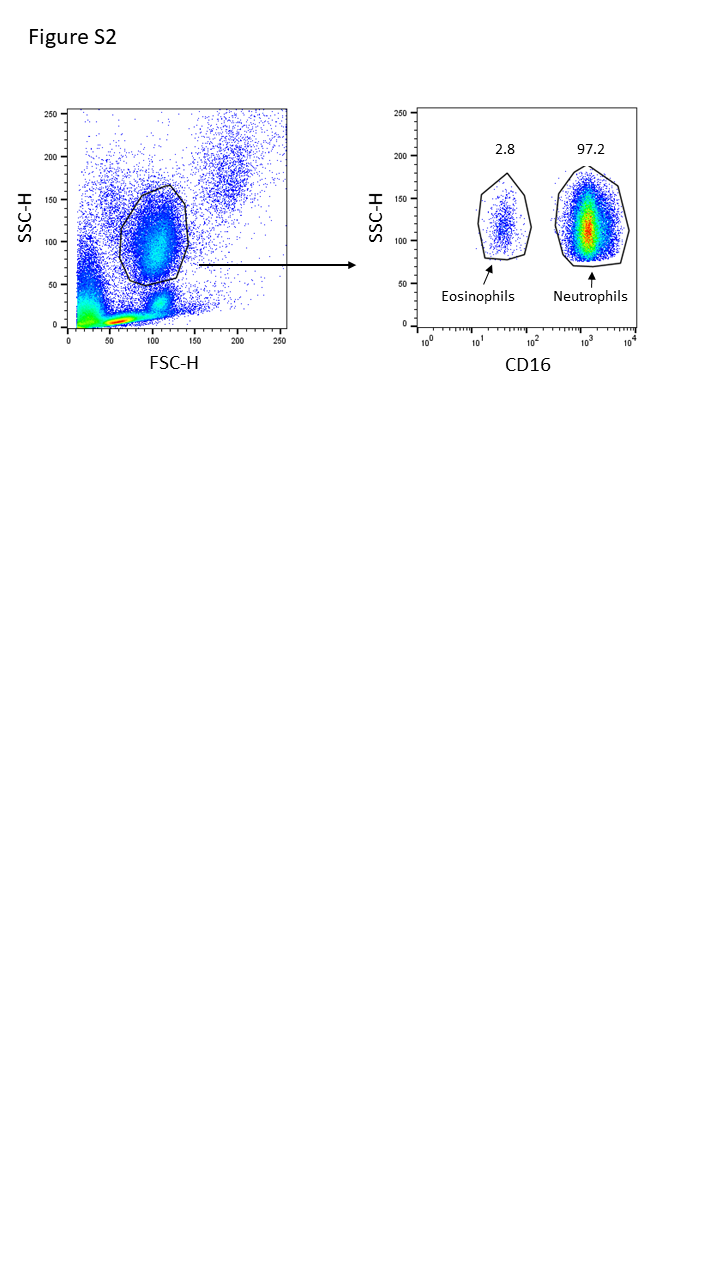

Supplement: Supplementary Figure 2 — Gating strategy to characterize proportions of peripheral blood neutrophils and eosinophils. Granulocytes were gated using forward- and side-scatter properties. Neutrophils were defined as CD16+ cells and eosinophils as CD16- cells. [file Image_2.tif]
